# On- or Off-pump Coronary Artery Bypass Surgery. Is the Debate Settling Down?

**DOI:** 10.21470/1678-9741-2019-0281

**Published:** 2019

**Authors:** Walter J. Gomes, Gianni D. Angelini

**Affiliations:** 1 Cardiovascular Surgery Discipline, Hospital São Paulo, Escola Paulista de Medicina, Universidade Federal de São Paulo, São Paulo, SP, Brazil; 2 Bristol Heart Institute, University of Bristol, Bristol Royal Infirmary, Bristol, United Kingdom.

The long-standing controversy on the merits and shortcomings of coronary artery bypass surgery (CABG) being performed on- and off-pump has been addressed by a recent series of robust evidences shedding light on prevailing disputed issues.

Long-term follow-ups from randomized trials using these two techniques are reassuring on the quality and safety of off-pump coronary artery bypass (OPCAB), therefore, bringing back a valuable tool for the coronary surgeon armamentarium.

## LONG-TERM OUTCOMES

The earlier concerns raised by the five-year follow-up of the Randomized On/Off Bypass (ROOBY) trial^[1]^, also by a 10-year analysis of a regional clinical registry in the United States of America^[2]^, suggesting an increased mortality and higher rate of graft failure in patients undergoing OPCAB than in those undergoing on-pump coronary artery bypass (ONCAB) were counterbalanced by a succession of well-conducted randomised controlled trials reporting long-term outcomes and demonstrating otherwise.

The Medicine, Angioplasty, or Surgery Study (MASS) III was the first study to reach the longest ever follow-up, 10 years, with 308 patients randomized; 155 OPCAB patients and 153 ONCAB patients. The endpoints were freedom from death, myocardial infarction (MI), revascularization, and cerebrovascular events. The 10-year follow-up revealed that event-free survival rates for ONCAB *vs*. OPCAB were 69.6% and 64% (hazard ratio [HR] 0.88; 95% confidence interval [CI] 0.86-1.02; *P*=.41), respectively. No difference was found between the groups in relation to primary composite endpoints at 10-year follow-up. Although OPCAB surgery was associated to a lower number of grafts and higher incidence of AF, it had no effects on long-term outcomes^[3]^.

The CORONARY trial randomized 4,752 patients to undergo off-pump or on-pump CABG. The five-year outcome analysed a composite outcome of death, stroke, MI, renal failure, or repeat coronary revascularization. No significant differences were seen between the off-pump group and the on-pump group in the rate of the composite outcome (23.1% and 23.6%, respectively) or in the rates of the components of the outcome, including repeat coronary revascularization. They concluded that the rate of the composite outcome of death, stroke, MI, renal failure, or repeat revascularization at five years of follow-up was similar among patients who underwent off-pump CABG and those who underwent on-pump CABG^[4]^.

The German Off-pump Coronary Artery Bypass Grafting in Elderly Patients (GOPCABE) trial enrolled 2,539 patients aged ≥ 75 years who were randomly assigned to undergo off-pump or on-pump CABG. The five-year follow-up data of this trial reported that 361 patients (31%) who assigned to off-pump CABG and 352 patients (30%) who assigned to on-pump CABG had died (HR off-pump/on-pump CABG 1.03; 95% CI 0.89-1.19; *P*=0.71). The composite outcome of death, MI, and repeat revascularization occurred in 397 patients (34%) after off-pump and in 389 patients (33%) after on-pump CABG (HR 1.03; 95% CI 0.89-1.18; *P*=0.704). Incomplete revascularization occurred in 403 (34%) patients in the OPCAB group and in 354 (29%) patients assigned to on-pump CABG (*P*<0.001). They concluded that, in elderly patients, ≥ 75 years of age, the five-year survival rates and the combined outcome of death, MI, and repeat revascularization were similar after on-pump and off-pump CABG. Incomplete revascularization was associated with a lower five-year survival rate, irrespective of the type of surgery^[5]^.

These results reinforce the long-term follow-up of several other studies. No difference in mortality was seen in the Optimising Cardiac Surgery ouTcOmes in People with diabeteS (OCTOPuS) trial after five years, the Beating Heart Against Cardioplegic Arrest Studies (BHACAS) I and II trials after eight years, or in the Surgical Management of Arterial Revascularization Therapies (SMART) trial after eight years of follow-up^[6-8]^.

## EXPERIENCE AS A DETERMINANT OF RESULTS - THE ROLE OF EXPERTISE

Mounting evidence demonstrated that outstanding outcomes with OPCAB have been associated with the surgeon’s and the team’s experience and expertise. In an analysis of 2,094,094 patients undergoing on- and off-pump CABG from the Nationwide Inpatient Sample database, OPCAB compared with on-pump CABG was associated with increased risk-adjusted mortality when performed in low-volume centers (< 29 cases/year) or by low-volume surgeons (< 19 cases/year). Conversely, in high-volume OPCAB centers (≥ 164 cases/year) and surgeons (≥ 48 cases/year), OPCAB reduced mortality compared with on-pump CABG in cases requiring a single graft or two or more grafts. Therefore, OPCAB outcome is dependent on volume at both the institution and the individual surgeon levels and should not be performed at low-volume centers and by low-volume surgeons^[9]^.

A post hoc analysis of the Arterial Revascularization Trial (ART) demonstrated that surgeons experienced with both on-pump and off-pump techniques, whether using single internal thoracic artery (ITA) or bilateral ITA grafts, yielded excellent results with no differences between the techniques, translated by low mortality, stroke, MI, and need for wound reconstruction and repeat revascularization^[10]^.

A recent large observational study demonstrated a reduction of mortality with off-pump compared with on-pump surgery, regardless of the number of grafts, if performed by experienced surgeons^[11]^.

## COST

A cost analysis of the two techniques had brought mixed results, but ultimately CORONARY and ROOBY trials demonstrated neutrality or higher costs incurred with OPCAB.

The substudy of the MASS-III trial, comparing the costs of the two techniques in Brazil, showed that OPCAB significantly decreased perioperative expenses, owing to a shorter orotracheal intubation time and length of stay in the intensive care unit, as well as reduction in the incidence of blood transfusions and perioperative MI. Such saving would result in an 25% increase in the availability for further care of surgical coronary patients^[12,13]^.

The study on the long-term cost-effectiveness of on-pump and off-pump CABG based on the MASS III trial estimated the healthcare resource usage over a five-year follow-up. Over a lifetime horizon, the incremental cost-effectiveness ratio of on-pump *vs*. off-pump CABG was US$12,576 per quality-adjusted life year (QALY) gained, which is above the suggested cost-effectiveness threshold range (from US$ 3,210 to 10,122), suggesting that on-pump CABG is not cost-effective when compared to off-pump CABG from a public health system perspective^[14]^.

Therefore, the contribution of these data is particularly relevant for the Brazilian healthcare systems, emphasizing the continued effectiveness and benefits of off-pump coronary revascularization and its lower comparative cost, with a resulting increase in the availability of the surgery for a larger number of patients. Both private and public health care systems may benefit from the reduction in costs, with no decrease in effectiveness.

## HIGH-RISK PATIENTS

Several analyses have suggested a beneficial effect of the OPCAB in selected subsets of high-risk and elderly patients, including those with left ventricular dysfunction, high calcific load, age > 75 years, diabetes, renal failure, left main stem disease, reoperations, chronic pulmonary disease, and an overall European System for Cardiac Operative Risk Evaluation (EuroSCORE) score of > 5. Potential advantages of off-pump surgery in these cohorts include reduction of the risk of death, stroke, and MI^[14,15]^.

A recent meta-analysis included 100 studies, with a total of 19,192 subjects and showed that OPCAB was associated with a significant 28% reduction in the odds of cerebral stroke (*odds ratio* [OR] 0.72; 95% CI 0.56-0.92; *P*=.009). A significant relationship between patient risk profile and benefits from OPCAB was found in terms of all-cause mortality (*P*<.01), MI (*P*<.01), and cerebral stroke (*P*<.01), suggesting that OPCAB should be strongly considered in high-risk patients^[16]^.

Worth of mention, diabetic patients account for the fastest growing cohort referred for surgical coronary revascularization, making 49% in the 2018 report of the Society of Thoracic Surgeons Adult Cardiac Surgery Database^[17]^. In-hospital adverse outcomes after CABG are more common in diabetic than in nondiabetic patients. Diabetes is both a marker for high-risk, resource-intensive, and expensive care after CABG and an independent risk factor for reduced long-term survival. Compared to nondiabetics, diabetics undergoing CABG present worse outcomes, with more in-hospital deaths, deep sternal wound infections, strokes, renal failure, prolonged postoperative hospital stay, and higher hospital costs^[18]^.

In a meta-analysis including 543,220 diabetic patients and comparing on- and off-pump outcomes, the overall mortality was comparable between the techniques, but OPCAB was associated with significantly decreased incidence of cerebrovascular events (OR 0.45; 95% CI 0.31-0.65; *P*<0.0001), an impressive 55% reduction^[19]^.

## THE CONUNDRUM OF LEFT MAIN CORONARY ARTERY DISEASE (LMCAD)

In the Evaluation of XIENCE Versus Coronary Artery Bypass Surgery for Effectiveness of Left Main Revascularization (EXCEL) trial, OPCAB use was more frequent than in the Synergy Between Percutaneous Coronary Intervention with TAXUS and Cardiac Surgery (SYNTAX) trial (29.6% *vs*. 15.4%). In a propensity-matched analysis of patients randomized to CABG in the SYNTAX and EXCEL trials, the composite endpoint of major adverse cardiac and cerebrovascular events at three years was higher in the SYNTAX group than in the matched population from the EXCEL trial (20.9% *vs*. 14.0%; *P*=0.008) The composite three-year endpoint of death, stroke, or MI was also higher in the SYNTAX trial than in the EXCEL trial (14.0% *vs*. 9.6%; *P*=0.05). Except for MI, all non-hierarchical components of the primary endpoint contributed to the better outcomes in the EXCEL trial compared with the SYNTAX trial: all-cause death (5.5% *vs*. 8.5%), any stroke (3.1% *vs*. 5.1%), and incidence density ratio (7.1% *vs*. 9.4%), respectively. Remarkably, the use of guideline-directed medical therapy was also greater in the EXCEL trial than in the SYNTAX trial^[20]^.

OPCAB, despite a lower number of grafts, was found to provide similar or superior outcomes in LMCAD compared to on-pump CABG, and smaller stroke rates, even employing manipulation of aorta with side-clamping^[21]^.

## EXPANDING THE CABG PROSPECT - THE AORTA NO-TOUCH (ANAORTIC) OPCAB TECHNIQUE

Stroke persists as the CABG’s Achilles’ heels in high-risk cohorts of coronary artery disease patients. CABG perioperative strokes have significant impact on length of hospital stay, incremental hospital resource consumption, and mortality outcome, with up to 10 times increase in hospital mortality rates^[22,23]^. Cardiopulmonary bypass is an independent risk factor for adverse neurologic outcomes^[24]^.

The anaortic OPCAB technique in the hands of highly trained teams has been demonstrated to reduce the risk of early stroke, by avoiding the ascending aorta manipulation and minimizing the potential for cerebral atheroembolic events. Some reports describe 0% early strokes after CABG with this technique, making this perioperative occurrence nearly virtual^[25-27]^.

The 2018 European Society of Cardiology/European Association for Cardio-Thoracic Surgery (ESC/EACTS) guidelines on myocardial revascularization unquestionably state that off-pump CABG and preferably no-touch techniques on the ascending aorta by experienced operators is Class I recommendation in patients with significant atherosclerotic aortic disease. Also, Class IIa is given to the technique for subgroups of high-risk patients. There is a special emphasis in patients with stable multivessel and/or LMCAD with porcelain aorta, where commonly the heart team recommendation is in favour of percutaneous coronary intervention, unless expertise exists with anaortic OPCAB. The guidelines recommend OPCAB in patients with renal impairment and suggest considering beating heart revascularization to reduce perioperative bleeding and the need for transfusions^[28]^.

## FINAL REMARKS

Forthcoming randomized trials should clarify the pending controversies related to the clinical application of OPCAB on the scenario above described. However, rather than antagonists, both techniques should be complementary to strength the coronary surgeon armamentarium, taking advantage of its potential benefits and a tailored best patient’s approach.

When performed by experienced surgeons in centres with adequate infrastructure, OPCAB is a safe alternative to ONCAB, regardless of the patients’ risk profile, and it is associated with reduction of hospital early complications and similar long-term outcomes.

OPCAB is a challenging technique requiring a steep “learning curve”. To master the learning curve, a team approach is of paramount importance. In this context, the emerging data suggest that additional benefit to patients can be obtained if the surgeon and the staff master the two techniques, and henceforth should be trained in both^[29,30]^.
